# Deep Learning for Cardiac Wall Motion Analysis: A Review of Methods, Challenges, and Clinical Applications

**DOI:** 10.1007/s10439-026-04147-0

**Published:** 2026-04-29

**Authors:** Mohammadali Monfared, Bahram Kakavand, Amirtahà Taebi

**Affiliations:** 1 Department of Bioengineering, Lehigh University, Bethlehem, PA 18015 USA; 2 Division of Pediatric Cardiology, Nemours Children’s Hospital, Orlando, FL 32827 USA

**Keywords:** Cardiac wall motion, Myocardial strain, Machine learning, Deep learning, Cardiac wall motion abnormalities

## Abstract

Abnormalities in cardiac wall motion are strong predictors of cardiovascular risk, making their accurate detection essential for early diagnosis and effective clinical management. Traditional imaging modalities such as echocardiography, magnetic resonance imaging (MRI), and computed tomography (CT) provide valuable insights but face limitations related to accessibility, cost, and the complexity of spatiotemporal analysis. Recent advances in machine learning (ML), particularly deep learning (DL), have enabled automated extraction of spatial and temporal features from medical imaging. They improved accuracy in segmentation, motion estimation, and detection of regional wall motion abnormalities. This paper reviews state-of-the-art methods for predicting cardiac wall motion, with emphasis on DL applications across echocardiography, 4D CT, and cine MRI datasets. Representative studies demonstrate the potential of convolutional neural networks, recurrent neural networks, and transformers to achieve performance comparable to expert clinicians, while also highlighting challenges such as data scarcity, model interpretability, and limited external validation. Addressing these issues will be critical for translating ML-based approaches into routine practice, and integration of advanced imaging with robust ML frameworks helps in developing a reliable cardiac wall motion simulators for personalized treatment planning and improved cardiovascular care.

## Introduction

Cardiac wall motion abnormalities (WMAs) are strongly associated with increased risks of cardiovascular events and mortality, with both their severity and spatial distribution serving as key prognostic factors. A landmark study demonstrated that adults without clinically evident heart disease but presenting with segmental or global WMAs had a 2.4- to 3.4-fold greater risk of future cardiovascular morbidity and mortality, independent of traditional risk factors [1]. Thus, WMAs represent a robust predictor of adverse cardiovascular outcomes and highlight the importance of cardiac wall motion analysis in early detection and clinical management.

Beyond their prognostic significance, cardiac wall motion studies contribute to understanding the biomechanical behavior of the myocardium under normal and pathological conditions [2]. The rhythmic contraction and relaxation of the heart rely on synchronized wall motion, and disturbances in this synchrony provide valuable diagnostic insight. For instance, late-contracting, non-contracting, or paradoxically contracting regions, particularly at the apex, are critical indicators of mechanical dyssynchrony and have been shown to predict patient response to cardiac resynchronization therapy [3]. Accordingly, systematic monitoring of cardiac wall motion is essential for accurate assessment of cardiac function and timely identification of pathological states.

**Fig. 1:**
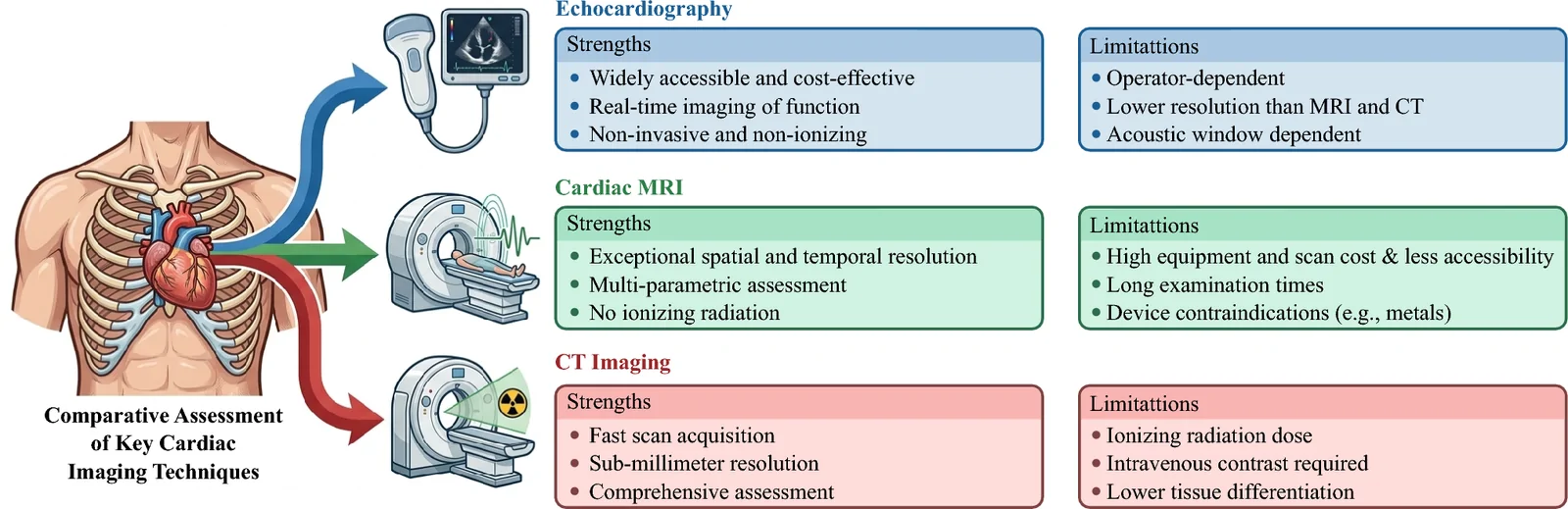
An overview of the current imaging modalities to capture cardiac motion

Imaging modalities facilitate systematic monitoring of cardiac wall motion and provide detailed visualization of myocardial mechanics [4]. Echocardiography, because of its real-time acquisition and accessibility, remains the most widely used tool for assessing wall motion, though it is limited by acoustic windows [5], which are specific anatomical pathways (typically intercostal spaces) where the ultrasound beam can reach the heart without being obstructed by the ribs (bone) or lungs (air). In practice, limited acoustic access results in the inadequate visualization of two or more myocardial segments (commonly in the lateral or apical regions), which impairs endocardial border delineation and reduces the accuracy of regional wall motion assessment and ejection fraction estimation [6]. Magnetic resonance imaging (MRI), particularly tagged MRI, offers high spatial and temporal resolution with superior reproducibility, which makes it the reference standard for quantifying myocardial deformation [7]. Computed tomography (CT) is an alternative for patients with contraindications to MRI. It enables high-resolution imaging with retrospective ECG gating, which allows reconstruction of multiple phases of the cardiac cycle [8]. Furthermore, to extract quantitative motion parameters across modalities and enhance the sensitivity and reproducibility of wall motion analysis, advanced computational approaches including feature tracking [9], optical flow [10], and speckle-tracking echocardiography [11] have been developed [12–17]. Figure 1 lists the main cardiac imaging methods to capture cardiac wall motions.

Despite these advances, conventional imaging and computational techniques often require labor-intensive preprocessing, manual annotations, or are limited in their ability to fully capture the spatiotemporal complexity of myocardial motion [4, 18–21]. Deep learning (DL) has recently emerged as a powerful alternative, capable of automatically extracting hierarchical features from large-scale imaging datasets and modeling non-linear dynamics with high accuracy [22–29]. DL-based approaches have been increasingly applied to cardiac imaging for tasks such as segmentation, motion estimation, and abnormality detection and provide the potential to overcome the limitations of traditional methods [4, 18, 30–33]. The primary objective of this study is to review the state-of-the-art methods for predicting cardiac wall motion, with a particular focus on the application of DL techniques to address existing challenges.

This review is organized as follows: we first establish the physiological basis of myocardial kinematics, then detail conventional deformation analysis methods. The core of the manuscript evaluates state-of-the-art DL models categorized by modality, including echocardiography as the clinical standard and high-resolution cine MRI and 4D CT. We conclude by advocating for a multimodal integration framework, particularly the fusion of CT’s spatial precision with echocardiography’s accessibility, as a primary path for future clinical translation.

## Methodology for Literature Identification

### Search Strategy and Information Sources

This review employs a structured narrative approach to synthesize recent methodological advances in DL (and in general machine learning, ML) for cardiac wall motion and strain analysis. To ensure a transparent and reproducible framework, the literature search was conducted following systematic principles. Primary literature searches were conducted through March 2026 across PubMed and Web of Science. Google Scholar was also used to leverage its comprehensive indexing of other databases including IEEE Xplore and Scopus.

Search queries were constructed using comprehensive Boolean logic to combine thematic keyword variants. The overarching search string utilized was: (“cardiac wall motion” OR “myocardial motion” OR “myocardial deformation” OR “myocardial strain” OR “myocardial mechanics” OR “myocardial kinematics” OR “ventricular motion” OR “ventricular deformation” OR “ventricular mechanics” OR “cardiac contraction” OR “myocardial strain” OR “radial strain” OR “circumferential strain” OR “longitudinal strain” OR “regional wall motion” OR “wall motion abnormalities”) AND (“cine MRI” OR “tagged MRI” OR “4D CT” OR “cardiac CT” OR “medical imaging” OR “speckle tracking echocardiography” OR “DENSE MRI” OR “echocardiography”) AND (“deep learning” OR “machine learning” OR “artificial intelligence” OR “convolutional neural network” OR “recurrent neural network” OR “long short-term memory”). This combinatorial approach ensured the capture of varying terminology for physiological and technical concepts. Forward and backward citation tracking was also employed to identify foundational methodological papers missed by keyword strings.

### Eligibility Criteria and Study Selection

**Fig. 2:**
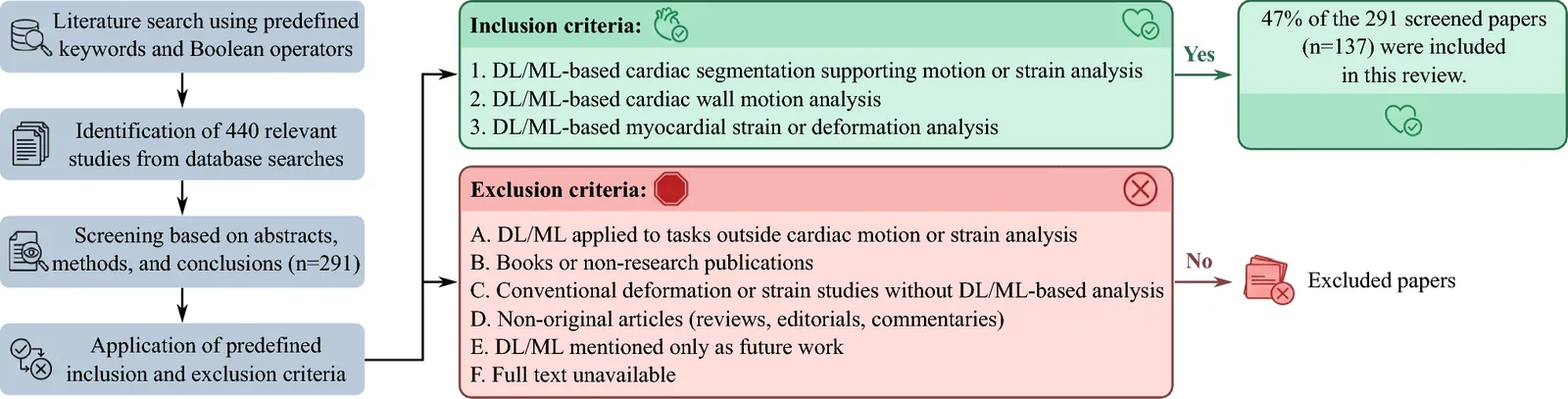
PRISMA-style flow diagram illustrating the identification, screening, and selection of relevant literature based on specific inclusion and exclusion criteria

Figure 2 illustrates the PRISMA-style flow diagram of the literature search and study selection process. The initial database search yielded 440 studies. Following deduplication, abstracts and titles were screened, resulting in the exclusion of 149 studies. The remaining 291 full-text articles were assessed against predefined inclusion and exclusion criteria. Studies were included if they: (1) proposed or validated DL/ML frameworks for cardiac segmentation supporting functional analysis; (2) performed DL/ML-based cardiac wall motion estimation; or (3) conducted DL/ML-based myocardial strain or deformation analysis. Studies were excluded if they applied DL/ML to tasks outside cardiac motion/strain, lacked clinical imaging data (purely theoretical simulations), were non-original publications (reviews, editorials), or mentioned DL/ML only as future work. Ultimately, 137 studies met all criteria and were included for qualitative synthesis.

Following the application of inclusion and exclusion criteria, the final pool of included studies was categorized based on their functional role within the cardiac analysis pipeline (Table 1). Initially, studies were grouped by singular tasks: DL-driven segmentation for downstream functional analysis, isolated wall motion and regional WMA classification, and standalone myocardial strain quantification. A qualitative synthesis of the recent literature reveals a pronounced shift toward integrated mechanics assessment. To reflect this, multi-component models were further subdivided into dual-task frameworks like combining segmentation with motion analysis, segmentation with strain estimation, or motion with strain quantification. Notably, the largest growing subset comprises comprehensive end-to-end DL and ML architectures that jointly perform segmentation, motion tracking, and strain analysis directly from native imaging, underscoring the field’s trajectory toward unified clinical tools.

**Table 1:** Categorization of included DL/ML studies by primary cardiac analysis application

Application focus	Representative methods	Target modalities	Included studies
Segmentation for Functional Analysis	U-Net variants, CNNs, Other architectures (e.g., DeepLabV3+, Temporal ConvNets)	Echocardiography, Cine and DENSE MRI, cardiac CT	[20, 21, 34–60]
Wall Motion and Regional WMA Analysis	CNNs, Other architectures (e.g., ANN, YOLO-based models, CoTracker, memory networks, transformers)	Echocardiography, Cine MRI	[22, 26, 61–74]
Myocardial Strain Quantification	RNNs, Traditional ML (e.g., SVM)	DENSE MRI, Echocardiography	[12, 14, 16, 28, 75–82]

Integrated and Multi-Task Frameworks			
Segmentation + Motion Analysis	Multi-task CNNs, registration-based DL, RNN-based models	Echocardiography, Cine MRI	[83–92]
Segmentation + Strain Estimation	CNNs, U-Nets, Other approaches, including ensemble learning, segmentation and speckle-tracking pipelines, and hybrid loss-based models	Echocardiography	[15, 17, 93–99]
Motion Analysis + Strain Quantification	CNNs, U-Nets, Optical flow-based DL (e.g., RAFT, PWC-Net, FlowFormer, TeeFlow, TeeTracker), Traditional ML	Echocardiography, Cardiac MRI	[100–116]
End-to-End Analysis *(Segmentation + Motion + Strain)*	CNNs, U-Net families, Transformer, Optical flow-based DL, Traditional ML (e.g., PCA)	Echocardiography, Cine and DENSE MRI, 4DCT	[4, 18, 19, 21, 23–25, 27, 28, 32, 33, 70, 117–143]

## Multidimensional Myocardial Kinematics

Building upon the clinical importance of studying WMAs, a comprehensive understanding of normal myocardial wall motion is essential to appreciate its physiological role in sustaining cardiac function and to recognize how deviations manifest in disease states.

The coordinated motion of the myocardium is fundamental to effective cardiac function, consisting of longitudinal, circumferential, radial, and torsional deformation patterns that together enable efficient blood ejection during systole and filling during diastole. These deformation patterns can be quantitatively described using myocardial strain, which captures the extent of motion in each direction and provides a standardized metric for assessing cardiac mechanics. Myocardial strain represents the degree of deformation, expressed as the percentage change in a myocardial segment’s length compared to its baseline length at end-diastole. Quantitative assessment of myocardial deformation, commonly referred to as strain analysis, provides a sensitive means to reveal changes in cardiac mechanics and facilitates the early detection, serial monitoring, and management of heart failure [144, 145]. To illustrate these motions, Table 2 summarizes the deformation patterns of the left ventricle (LV) during the cardiac cycle together with representative normal quantitative ranges reported for healthy adults. The reported peak-systole strain and twist values are relative to end-diastole, which served as the zero-deformation reference state.

**Table 2:** Myocardial deformation of left ventricle (LV) during systole and diastole in healthy adults

Direction/mechanics	Systolic phase	Diastolic phase	Normal ranges at peak systole [146]
Longitudinal	Shortening	Lengthening	Endocardial peak systolic strain: − 19.6 ± 2.0%
			Base-to-apex increase 10%
Circumferential	Shortening	Lengthening	Endocardial: − 27.6 ± 3.9%
			Epicardial: − 11.3 ± 2.2%
			Base-to-apex increase 33%
Radial	Thickening	Thinning	Endocardial thickening: + 30.1 ± 7.5%
			Apical thickening 16% lower than base
Twist Mechanics	LV twisting	LV untwisting	Endocardial twist: 9.6 ± 3.9$^{\circ}$
			Epicardial twist: 4.0 ± 1.9$^{\circ}$

**Fig. 3:**
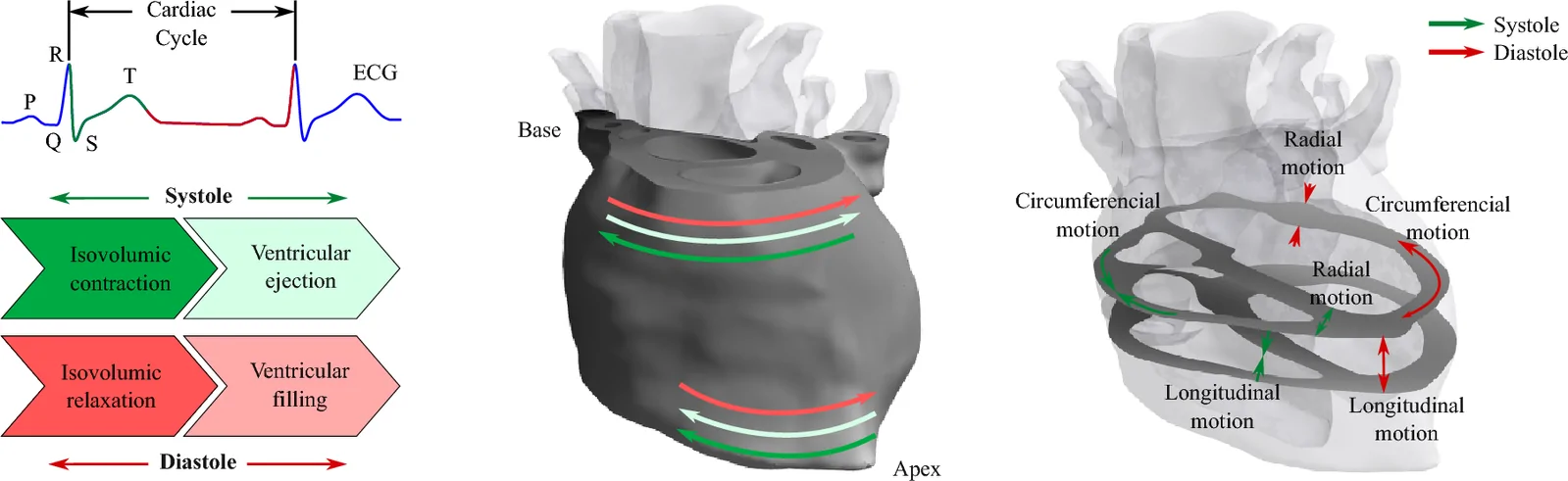
Schematic representation of cardiac wall motion and twist mechanics throughout the cardiac cycle. Blue arrows indicate torsions during isovolumic contraction. Green arrows represent motions and torsions during systole, where the myocardium shortens longitudinally and circumferentially, thickens radially, and the apex rotates counterclockwise while the base rotates clockwise, producing a wringing motion for efficient ejection. Yellow arrows denote torsions during isovolumic relaxation, during which torsional deformation is rapidly released as the apex recoils clockwise, initiating early diastolic suction. Red arrows correspond to motions and torsions during diastole, when the myocardium lengthens and thins, and ventricular untwisting completes, enabling rapid filling

Longitudinal shortening and circumferential contraction are the principal contributors to ventricular ejection, while radial thickening supports chamber emptying by increasing wall thickness and reducing cavity volume. In addition to these motions, twist mechanics are determined by the helical arrangement of myocardial fibers. Much like wringing a towel, the apex and base rotate in opposite directions during the cardiac cycle. When viewed from the apex, the apex rotates counterclockwise and the base rotates clockwise during systole, contributing to efficient blood ejection. This torsional deformation is then rapidly released during isovolumic relaxation as the apex recoils clockwise, generating early diastolic suction and completing untwisting in early filling [147, 148]. Beyond intrinsic mechanics, respiration introduces cardiac motion that is primarily dominated by bulk translation (predominantly along the superior–inferior axis), though it also induces minor non-rigid deformations [149, 150]. During deep inspiration, the heart can shift significantly (up to 20+ mm), while localized non-rigid displacements, particularly in the right atrial and left ventricular free walls, remain modest (typically <7 mm) [151–153]. Crucially, this respiratory motion acts as a significant confounding factor in wall motion assessment; global translation can be misidentified as local myocardial deformation, leading to artifacts or “pseudo-dyskinesis.” Consequently, distinguishing these measurement-level shifts from true physiological contraction is fundamental to the reliable characterization and clinical interpretation of regional wall motion abnormalities. Figure 3 indicates the schematic representation of cardiac wall motion and twist mechanics throughout the cardiac cycle.

## Myocardial Deformation Analysis

While imaging modalities such as echocardiography, MRI, and CT provide the raw spatiotemporal data of cardiac motion, the evolution of myocardial deformation analysis has focused on extracting quantitative metrics from these images to characterize ventricular mechanics accurately. Technological progress has enabled the transition from indirect indices of ventricular function toward direct assessment of myocardial deformation. Early methods such as the 1D tissue Doppler technique [154–156] evolved into 2D speckle tracking echocardiography [157, 158], which in turn paved the way for the application of speckle tracking technology to 3D echocardiographic datasets [159, 160]. This development has been critical for capturing the inherently three-dimensional nature of cardiac motion.

Recent advancements in ML have significantly enhanced the analysis of cardiac wall motion, which leads to improved diagnostic accuracy and patient outcomes. In this context, supervised learning has been employed to train models on labeled datasets for predicting outcomes such as myocardial strain or detecting regional WMA, while unsupervised learning enables the discovery of latent structures and clustering of motion patterns without prior labels. DL methods, particularly convolutional neural networks (CNNs) and recurrent neural networks (RNNs), have shown exceptional promise for modeling the spatiotemporal dynamics of cardiac motion [161, 162]. Recent studies have demonstrated that DL models can automatically detect and localize regional WMA from standard echocardiographic cine images with diagnostic accuracy comparable to expert cardiologists [61–63, 67, 73, 102, 163, 164].

## Linking Myocardial Motion to Clinical Outcomes

It is important to consider how alterations in the mechanical processes of the heart manifest in pathological conditions such as heart failure. Pathophysiological processes underlying heart failure lead to multiple mechanical consequences. This includes alterations in the 3D geometry of cardiac chambers, changes in ventricular wall thickness, and disruptions in both contractile function and relaxation. When these mechanisms are impaired, myocardial deformation becomes compromised [165, 166]. Depending on the underlying etiology and severity, deformation abnormalities may present regionally or globally, with changes in magnitude, timing, and rate.

Both congenital and acquired abnormalities of the heart can impair its function, disrupting the processes of filling and ejection and ultimately resulting in heart failure [167]. One hallmark of pathological remodeling is hypertrophy of cardiomyocytes, which frequently coincides with contractile dysfunction and enlargement of ventricular chambers, particularly in systolic heart failure [168]. Additionally, remodeling involves both cardiomyocyte and non-cardiomyocyte components and they produce mechanical consequences that manifest as alterations in the 3D deformation of the myocardium [169].

## Deformation Analysis Methods

Myocardial deformation analysis has evolved considerably over the past two decades, progressing from indirect indices of ventricular performance to advanced imaging techniques capable of directly quantifying strain. Tissue Doppler imaging [170], two-dimensional speckle tracking echocardiography [171], cardiac magnetic resonance feature tracking [171], three-dimensional wall motion tracking [170], and tagged cardiac MRI [172] have emerged sequentially, each aiming to overcome the limitations of its predecessors. However, every technique presents its own strengths and weaknesses. A summary of their respective advantages and disadvantages is provided in Table 3.

**Table 3:** Comparison of myocardial deformation analysis methods

Method	Strengths	Weaknesses
Tissue Doppler Imaging (TDI)	High temporal resolution Enables LV torsion assessment	Angle-dependent One-dimensional only Noisy signals, poor reproducibility Confined to ultrasound beam motion
2D Speckle Tracking Echocardiography (2D-STE)	Largely angle-independent Quantifies longitudinal, circumferential, radial strain Measures LV twist mechanics Widely available	Out-of-plane motion artifacts Limited to 2D analysis Foreshortened views reduce accuracy Separate apical/basal acquisitions needed
3D Wall Motion Tracking (3D-STE)	True 3D strain (longitudinal, circumferential, radial) Measures endocardial area strain, twist, dyssynchrony Single full-volume acquisition Better correlation with cardiac MRI	Lower spatial and temporal resolution Requires high-quality images Vendor/software variability More computationally intensive
Cardiac MRI Feature Tracking (cardiac MR-FT)	Uses standard cine cardiac MRIs Not limited by acoustic window Provides global and regional strain Good reproducibility	Lower temporal resolution than echo Higher cost and limited availability Longer acquisition and processing Less validated clinically
Tagged cardiac MRI	Gold standard for strain analysis Accurate and reproducible Captures full 3D and transmural strain	Requires special tagging sequences Long acquisition time Labor-intensive post-processing Limited clinical use

## Machine Learning Models for Predicting Cardiac Motion

ML has transformed cardiac motion prediction by enabling the analysis of complex, high-dimensional data and providing robust solutions for identifying subtle motion patterns. Among the ML techniques employed, CNNs, RNNs, and transformers have emerged as key models in this domain.

CNNs are particularly effective in analyzing spatial data, such as medical images, and extracting hierarchical features related to cardiac structure and motion [173, 174]. CNN architectures, such as U-Net, have been successfully applied to segment myocardial regions and predict motion abnormalities [36, 44, 63, 73, 175]. For instance, Beevi et al. [175] developed an automated DL model using U-Net for LV segmentation, followed by a 3D CNN for detecting regional WMAs in echocardiographic videos. This approach achieved high accuracy, precision, recall, and *F*1 scores, highlighting the potential of U-Net in cardiac motion analysis [175].

RNNs, including long short-term memory (LSTM) networks, excel at modeling temporal dependencies and are commonly used for time-series data, such as kinematic or ECG-derived cardiac signals [34, 40, 57, 176]. Recent advancements have also integrated RNNs with CNNs to leverage both spatial and temporal features in cardiac motion prediction [84, 177]. A study by Zhang et al. [34] introduced a multi-level convolutional LSTM model for segmenting the LV myocardium in infarcted porcine cine MRIs. The study results suggested that this approach was effective in capturing spatial and temporal dynamics, and enhancing segmentation accuracy in the presence of myocardial infarction [34].

Transformer-inspired co-attention mechanisms may improve unsupervised motion tracking and strain estimation in 3D echocardiography which demonstrates their ability to capture inter-frame spatial and temporal dependencies and enhance motion field regularization in noisy ultrasound data [178].

Although these ML models have enabled breakthroughs in automating cardiac motion analysis and reduce reliance on manual interpretation, challenges remain, including the need for large annotated datasets, model interpretability, and clinical validation. Table 4 summarizes representative DL studies, highlighting dataset size, validation strategy (internal vs. external), and reported clinical or technical performance metrics such as Dice coefficients, segmentation accuracy, or strain error.

**Table 4:** Comparative summary of representative deep learning studies for cardiac wall motion and myocardial deformation analysis

Modality	Dataset (*N*)	Validation type	Key performance metrics
Computed Tomography (CT)			
4D ECG-gated CT [179]	100 Patients	Internal (Single-center)	WMA Detection: Accuracy 83%; Sensitivity 63.2–81.3%; Specificity: 87.1–96.4%. Strain: $R^{2}$ = 0.91–0.92 vs. FAC
4D CT [164]	343 Patients	Internal (Single-center)	WMA Detection: Accuracy 93.5%; Sensitivity 91.9%; Specificity 94.7%; Cohen’s κ 0.87
4D Contrast CT [29]	1509 Patients	Internal (Single-center)	Segmentation: Dice 0.89 ± 0.10; ASSD 1.4–1.5 mm; 98.5% clinically usable LV segments
ECG-gated CT [58]	71 Patients	Internal (Single-center)	Segmentation: Overall Dice 0.920; Myocardium Dice 0.93; Volume correlation *r* 0.90–0.99
Cardiac Magnetic Resonance Imaging (MRI)			
Cine MRI (SSFP) [177]	219 Patients	Internal (Single-center)	WMA Detection: AUC 0.90; Accuracy 86%; Sensitivity 86%; Cohen’s κ 0.60–0.78
Cine MRI + DENSE [13]	305 Subjects	Internal (Multi-center)	Strain: Displacement EPE 0.75 ± 0.35 mm; Circumferential ICC 0.87; Correlation *r* 0.88
Cine MRI [176]	299 Subjects	Internal (Single-center)	MI Detection: Segment-level AUC 0.94; Sensitivity 89.8%; Dice vs. LGE 86.1 ± 5.7%
Tagged MRI [180]	23 Subjects	Internal (Single-center)	Motion Tracking: RMS error 1.63 ± 0.59 mm; Confirmed Diffeomorphic deformation

Studies are evaluated by dataset scale, validation type, and technical/clinical performance metrics

### Cardiac Wall Motion Extraction from 4D CT Images

The integration of DL with 4D cardiac CT has transformed how myocardial motion and regional WMAs can be quantified. Unlike conventional strain analysis methods that rely on manual segmentation and tracking, DL frameworks are now capable of learning temporal and spatial patterns of cardiac deformation directly from ECG-gated CT data, enabling more reproducible and automated assessment of ventricular function.

Recent studies demonstrate that DL-based segmentation and motion quantification pipelines can reliably extract dynamic information from 4D CT volumes with accuracy comparable to expert interpretation [164, 177]. CNNs and their 3D variants have shown particular promise for whole-heart and LV segmentation, achieving Dice similarity coefficients above 0.85 even in large and heterogeneous clinical datasets [29, 58]. Such models can delineate cardiac structures across multiple cardiac phases, providing the foundation for downstream computation of regional strain and motion metrics. Despite these advances, segmentation robustness still declines in cases of low contrast enhancement or motion artifacts, underscoring the need for domain adaptation and artifact-aware architectures [29].

Beyond segmentation, several studies have proposed DL strategies that directly estimate myocardial deformation or classify WMAs from 4D CT sequences. These methods often combine CNN-based feature extraction with temporal modeling using recurrent or transformer-based networks to capture the cyclical nature of cardiac motion [164, 179]. This approach allows efficient data compression, sometimes reducing 4D CT datasets by orders of magnitude, while preserving clinically relevant motion signatures [164]. Reported accuracies exceeding 90% for WMA detection highlight the potential of these architectures to support rapid, fully automated functional assessment in routine CT angiography [164, 179].

Nevertheless, current DL-based motion estimation remains constrained by key limitations. Most algorithms rely predominantly on endocardial boundary motion, neglecting wall thickening and transmural strain, which are important indicators of contractile dysfunction [179]. Training datasets are typically derived from single-center studies with limited patient diversity [58, 179], and ground-truth labeling often depends on subjective expert interpretation rather than quantitative gold standards. Moreover, binary classification of WMA does not capture the full spectrum of regional dysfunction severity, limiting clinical interpretability [164].

**Fig. 4:**
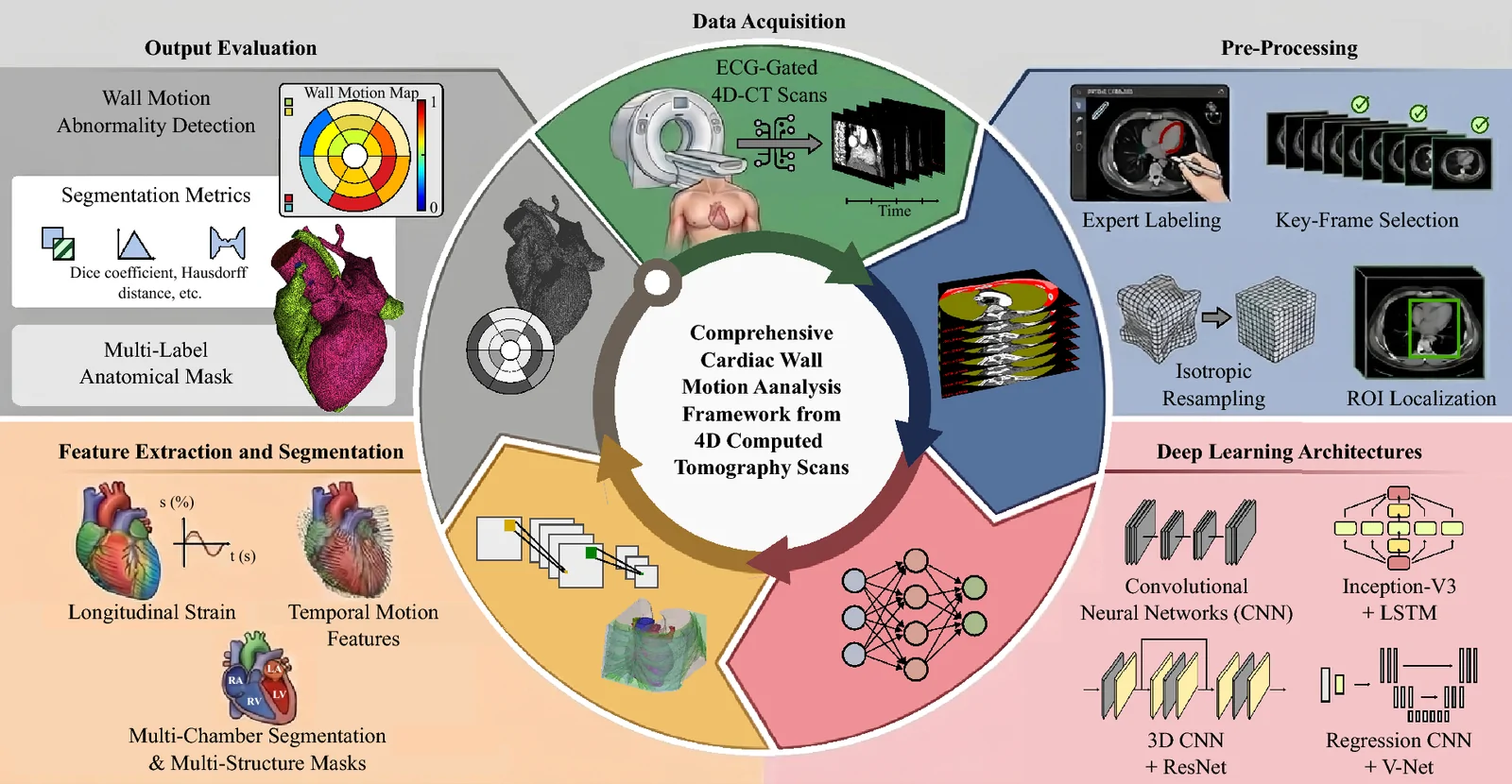
DL framework for cardiac wall motion analysis from 4D CT images, synthesized from studies identified through the literature identification strategy described in Sect. 2. The schematic illustrates common pipeline components, including ECG-gated data acquisition, preprocessing, CNN-based segmentation, temporal feature extraction, and downstream motion or wall-motion abnormality analysis

Looking forward, integrating biomechanical priors and physics-informed loss functions may enhance physiological plausibility and generalizability of DL-based motion tracking. Coupling 4D CT–derived strain fields with complementary modalities such as echocardiography or cardiac MRI could further improve validation and enable multimodal learning [92, 181]. Ultimately, these developments will advance DL-enabled 4D CT from a primarily research-oriented tool toward a clinically deployable solution for comprehensive myocardial mechanics assessment [112, 123, 134]. Figure 4 summarizes representative DL frameworks to analyze cardiac wall motion from CT images.

### Capturing Cardiac Wall Motion from Cine MRI

DL approaches have also been extensively applied to cine MRI, which provides high spatial and temporal resolution for assessing myocardial deformation and detecting regional WMAs with reduced observer variability. Compared to traditional feature-tracking or tagging methods, DL frameworks can extract dense motion fields and strain parameters directly from image sequences, enabling automated, vendor-independent quantification of myocardial mechanics.

Several architectures have been developed to leverage the rich temporal dynamics in cine MRI for myocardial motion estimation [25, 85, 122]. Masutani et al. [177] introduced a DL synthetic strain framework that simultaneously performs myocardial segmentation and velocity field estimation from short-axis cine steady-state free precession images [177]. Using a modified 3D U-Net, their model produced strain and strain rate maps that captured both radial and circumferential deformation, achieving diagnostic accuracy comparable to expert readers (AUC = 0.90). The results of this work suggest that CNN-based spatiotemporal modeling can provide clinically reliable WMA detection, although generalization remains limited by single-vendor training data and the lack of validation against prospective strain-imaging modalities.

To further enhance strain estimation fidelity, Wang et al. [13] proposed a CNN model trained using displacement-encoding with stimulated echoes (DENSE) data as ground truth [13]. The model learned to infer displacement fields from standard cine MRI inputs, achieving higher reproducibility and lower error than conventional feature tracking across both global and segmental circumferential strain measurements. This model represents an important step toward bridging the gap between DENSE precision and routine cine imaging availability. Nonetheless, reduced accuracy in basal regions and the absence of external validation suggest that robust cross-domain generalization remains a key challenge.

Beyond deformation quantification, DL methods have also been employed for direct functional and diagnostic classification from cine MRI. For example, Zhang et al. [176] developed a hybrid CNN–RNN architecture that localized the LV, extracted motion features via LSTM units and optical flow, and classified chronic myocardial infarction versus normal myocardium [176]. Their approach achieved strong diagnostic performance (AUC = 0.94), comparable to late gadolinium enhancement imaging, underscoring the potential of motion-based DL models to identify infarcted tissue without contrast agents. However, the method’s dependence on manually delineated training labels and single-center data underscores the need for more diverse and fully automated training pipelines.

As research progressed from functional quantification toward outcome prediction, ML frameworks began leveraging cardiac motion patterns to infer patient prognosis [37, 69]. Dawes et al. [182] provided one of the earliest demonstrations of this approach by analyzing three-dimensional right ventricular motion from cine cardiac MRI in patients with pulmonary hypertension [182]. Using a supervised principal-component model, they identified spatially distinct motion signatures, particularly reduced basal longitudinal and septal radial motion, that were strongly associated with mortality. They achieved an AUC of 0.73 for 1-year survival. This study set a foundation for prognostic motion-based phenotyping for subsequent DL–driven survival modeling.

To advance the ability to detect and classify regional dysfunction, Bello et al. [183] linked cardiac motion analysis to patient prognosis [183]. Using cine MRI sequences from individuals with pulmonary hypertension, they developed a fully convolutional segmentation pipeline coupled with a supervised denoising autoencoder (4D-Survival) that learned latent motion representations optimized for survival prediction. By integrating motion-derived features with a Cox partial-likelihood loss, the network achieved higher concordance (C=0.75) than conventional volumetric or strain-based indices. It suggests that dynamic cardiac motion fields can serve not only for functional quantification but also as independent predictors of patient outcome. The framework remained constrained by its reliance on manually labeled training data and internal validation from a single cohort that can be improved by adding external validations with unsupervised or self-supervised strategies capable of learning motion dynamics without explicit annotations.

Complementary to supervised methods, unsupervised and self-supervised approaches are emerging to overcome annotation constraints. These approaches are now enabling cardiac motion models to leverage large unlabeled imaging datasets [59, 84, 86, 129]. Self-supervised learning strategies for cardiac MRI segmentation involves predicting automatically defined anatomical positions from standard cardiac view planes [184]. This approach leverages the vast unannotated structural information inherent in cardiac MRI datasets to learn robust image representations and thereby improves segmentation accuracy in data-scarce settings [59]. For instance, Ye et al. [180] introduced a bi-directional generative diffeomorphic registration framework for motion tracking in cardiac tagging MRI [180]. By composing inter-frame motion fields through a differentiable layer, the method achieved dense and temporally consistent tracking of myocardial deformation across the cardiac cycle. Such work indicates that DL might be used to learn physically meaningful motion representations without explicit ground-truth labels, though accuracy can be affected by smoothness regularization and challenges in capturing rapid early-systolic motion.

Beyond data-driven architectures, physics-informed and geometry-aware frameworks further integrate mesh-based or fiber-oriented representations to align 2D image sequences with 3D cardiac geometries and embed biomechanical constraints and anatomical priors into the learning process. This will improve interpretability and cross-modal generalizability of motion estimation [185]. This structure-aware formulation enables physiologically coherent deformation tracking and facilitates population-level analysis of myocardial motion and shows a transition from intensity-based to anatomy-based DL models for cardiac mechanics assessment [16, 88, 107]. For instance, Meng et al. [186] introduced a geometry-consistent framework that models cardiac motion directly on a 3D mesh representation of the myocardium [186]. By integrating a differentiable mesh-to-image rasterizer, the model jointly reconstructs end-diastolic meshes and estimates vertex-wise 3D motion fields from multi-view cine MRI while preserving anatomical topology and vertex correspondence across cardiac phases. Furthermore, physics-informed neural networks can enforce myocardial incompressibility and fiber-aligned contraction within the loss function to produce physiologically consistent deformation fields from cine MRI [187].

**Fig. 5:**
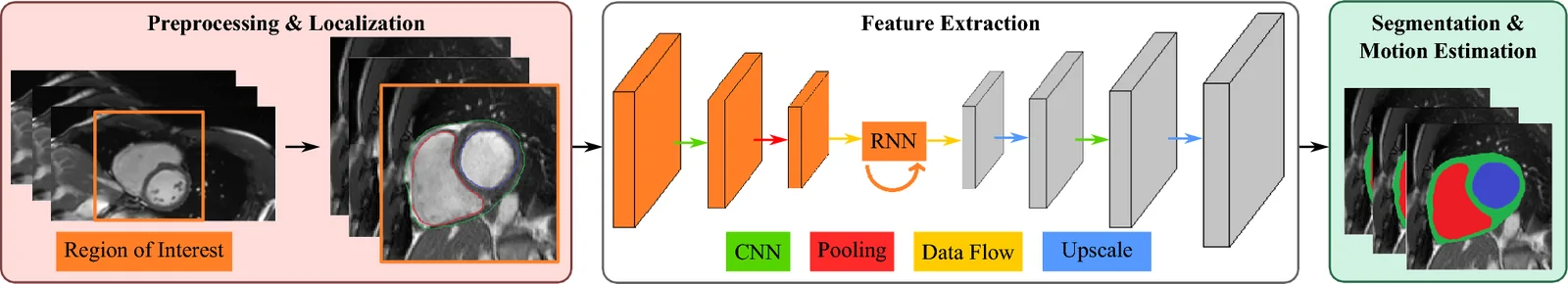
DL pipeline for segmentation and myocardial motion estimation from cine MRI, reflecting methodological patterns reported in studies identified through the keyword-based literature search. The framework highlights typical stages of localization, spatiotemporal feature extraction, and motion or strain estimation

Collectively, these studies highlight the growing maturity of DL-enabled cine MRI analysis, from segmentation-based strain mapping to direct motion-field estimation and pathology classification. Current limitations include dependence on vendor-specific training data, lack of standardized motion ground truth, and variable accuracy across myocardial regions. Future directions involve incorporating biomechanical constraints and physics-informed learning to improve physiological plausibility, integrating multimodal datasets such as DENSE and tagging MRI for hybrid supervision, and extending analyses to three-dimensional strain and torsion. Such developments will help realize the full potential of DL-based cine MRI for comprehensive, automated myocardial mechanics assessment. Table 5 summarized the general framework for cine MRI-based DL cardiac wall motion analysis with representative studies. Figure 5 schematically shows a sample DL framework for segmenting and capturing cardiac wall motion using cine MRI.

**Table 5:** General framework for cine MRI-based DL cardiac wall motion analysis with representative studies

Step	Description	Representative studies
Image acquisition	Obtain cine MRI sequences (e.g., short-axis cine or tagging MRI) covering the full cardiac cycle	[13, 176, 177, 180]
Preprocessing and localization	Perform segmentation or localization of the LV to define regions of interest and extract myocardial contours	[13, 176]
Feature extraction	Apply DL encoders (e.g., 3D U-Net, CNN) to capture spatiotemporal motion features; integrate temporal modeling techniques such as RNNs/LSTMs or optical flow for global/local motion characterization	[13, 176, 177]
Motion/displacement estimation	Predict myocardial velocity or displacement fields, either supervised (using ground-truth from DENSE or tagging) or unsupervised (via deformable registration networks)	[13, 177, 180]
Post-processing	Compute derived measures such as strain and strain-rate maps, decomposed into radial, circumferential, and/or longitudinal components	[13, 177]
Classification/diagnosis	Use extracted motion features to classify pathological states (e.g., myocardial infarction vs. normal tissue, detection of wall motion abnormalities)	[176, 177]
Evaluation and validation	Compare predicted motion/strain metrics against clinical ground truth using metrics such as AUC, sensitivity, specificity, accuracy, and reproducibility	[13, 176, 177]
Considerations	Account for challenges such as vendor-specific training data, reduced accuracy in basal sections, lack of longitudinal/radial strain analysis, dependency on manual annotations, and limited generalizability to external datasets	[13, 176, 177, 180]

### DL-Based Myocardial Deformation Analysis in Echocardiograms

While 4D CT and cine MRI have dominated research on DL-based cardiac wall-motion quantification, echocardiography remains the most ubiquitous imaging modality in clinical practice. Its real-time, bedside availability and affordability make it an indispensable tool for functional cardiac assessment. Beyond task-specific developments, DL in echocardiography is evolving toward a fully integrated clinical ecosystem, where automated image acquisition, view recognition, segmentation, and motion quantification operate within a unified workflow [14, 38, 181]. Therefore, complementing CT/MRI-centric studies with a dedicated overview of DL approaches for echocardiographic motion tracking is essential to bridge the gap between lab-based innovation and routine clinical deployment [181].

Early developments integrated statistical motion priors with deep classifiers to track endocardial boundaries in noisy ultrasound data [188]. Building on these preliminary hybrid frameworks, subsequent work achieved fully automated and clinically scalable view classification in echocardiographic videos to establish the foundation for downstream motion and functional analysis [73, 189]. Recent studies develop fully automated echocardiographic pipelines encompassing view selection, quality control, segmentation, regional WMA detection, and cardiac function quantification to extending DL applications from controlled research settings to real-world clinical environments. Unlike earlier models trained on static or high-quality datasets, some of these frameworks are validated on more than 7000 studies from both standard and portable bedside ultrasound systems, achieving consistent diagnostic accuracy (AUC ≈ 0.9) and demonstrating the feasibility of DL-based wall-motion assessment for clinic usages [190].

**Fig. 6:**
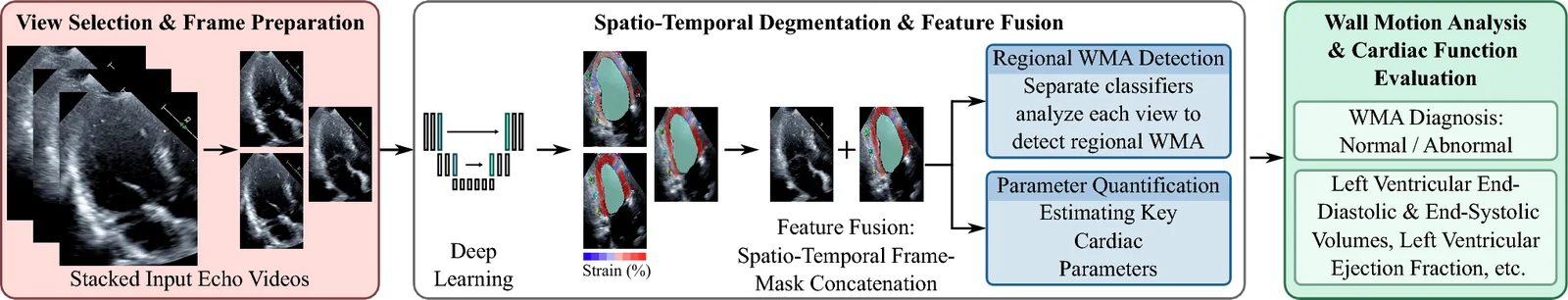
Representative schematic of a DL-based echocardiographic workflow synthesized from the reviewed literature. The pipeline illustrates commonly reported stages of view selection, spatiotemporal segmentation, wall-motion analysis, and functional quantification as identified through the literature identification strategy

A representative schematic of a general DL–based echocardiographic workflow is shown in Fig. 6. It illustrates the process of integrating view selection, spatiotemporal segmentation, wall-motion analysis, and functional quantification (e.g., left ventricular ejection fraction analysis) within a unified DL pipeline.

### Validation Strategies

Validation is a critical step in ensuring the reliability and clinical applicability of ML models for predicting cardiac motion. Methods for validation typically include cross-validation on labeled datasets, external validation using independent test sets, and model performance benchmarking against established metrics like root mean square error (RMSE) for displacement, Dice coefficient for segmentation accuracy, and area under the receiver operating characteristic curve for classification tasks [161, 174]. Additionally, advanced techniques such as *k*-fold cross-validation and bootstrap resampling are employed to assess model robustness and prevent overfitting. Employing bootstrap-based internal validation represents a robust strategy for assessing model stability and estimating optimism-corrected performance. This approach enables reliable evaluation of predictive generalizability without requiring large external cohorts. For example, Bello et al. demonstrated its effectiveness in a survival-prediction framework, where combining bootstrap resampling with Harrell’s concordance index yielded consistent accuracy (C=0.75) and minimized overfitting [183].

A rigorous validation approach involves repeated cross-validation combined with parameter tuning. For example, Zhang et al. [191] separated training, tuning, and testing datasets and applied 10-times repeated 10-fold cross-validation to reduce bias and limit overfitting. Each iteration stratified the dataset into 10 folds of equal size: eightfolds (80%) for training, onefold (10%) for hyperparameter tuning, and onefold (10%) for testing. Stratification ensured that each fold preserved a similar distribution of the data. This procedure was repeated across 10 iterations, rotating the folds used for training, tuning, and testing. The DL scores from all testing folds were concatenated to evaluate performance across the entire dataset. Model performance was then assessed by averaging AUC and accuracy across repetitions, enabling a fair comparison between DL predictions and human reader diagnosis.

Comparisons with experimental or clinical data are also essential to bridge the gap between computational predictions and real-world applicability. For instance, ML-derived strain measurements are often validated against gold-standard imaging techniques like tagged MRI or speckle-tracking echocardiography [192]. In an external validation on three independent cohorts, including a real-world population, a core-lab measured HFpEF dataset, and patients with suspected myocardial infarction; a DL-based algorithm achieved strong agreement with manual strain measurements (bias ≤ 0.7%, RMSE 2.6–2.8, correlation r≥0.76) and good discrimination for both global and regional wall-motion abnormalities (AUCs 0.80–0.98) [99]. Another validation approach applied to echocardiography datasets (995 exams total) demonstrated intra-class correlation coefficients of 0.87 internally and 0.80 externally, mean absolute errors of 0.33–0.35 cm in width measurements, and high AUCs (0.93 internal, 0.84 external) for detecting pericardial effusion, thereby confirming robust generalizability and agreement with expert human measurements [193]. Furthermore, experimental studies using in vivo or ex vivo cardiac models provide a means to compare predicted wall motion against direct measurements of myocardial mechanics. Clinical validation through prospective studies ensures that ML models can generalize across diverse populations, making them suitable for real-world deployment [57]. By combining computational, experimental, and clinical validation approaches, including repeated cross-validation with hyperparameter tuning, more reliable and interpretable ML-based systems for cardiac motion prediction can be built.

## Open Issues and Areas for Future Investigation

### Multimodal Fusion: Bridging the Gap Between CT and Echocardiography

While individual modalities offer unique strengths, the future of robust wall motion analysis lies in the synergy of multimodal datasets through DL. A primary area for future investigation is the integration of high-resolution 4D CT spatial data with the real-time clinical accessibility of echocardiography. Currently, 4D CT provides superior anatomical detail and 3D geometric “ground truth,” yet its use is limited by radiation exposure and cost. Conversely, echocardiography is the clinical mainstay but suffers from suboptimal acoustic windows and lower spatial resolution.

By leveraging cross-modality DL frameworks, researchers can use high-fidelity CT datasets to train models that learn complex 3D deformation signatures. These pre-trained features can then be adapted via transfer learning or distillation to enhance motion tracking in noisier echocardiographic cine images. In other words, the objective of multimodal fusion is not to mandate concurrent CT and echocardiographic imaging in routine practice, which would be cost-prohibitive. Rather, it is to leverage high-fidelity 4D CT datasets during the model development phase to serve as a ‘silver standard’ for training. Such a multimodal approach would allow for the “high-resolution” insights of CT to be deployed at the bedside, fundamentally improving the sensitivity of echocardiography for detecting subtle regional wall motion abnormalities without requiring additional advanced imaging for every patient.

### Comparative Analysis and Critical Synthesis of DL Architectures

While convolutional, recurrent, and attention-based architectures have all demonstrated utility in cardiac motion analysis, their performance is strongly influenced by the spatiotemporal characteristics of cardiac imaging data and the underlying biomechanics of myocardial deformation. CNN-based models are effective for spatial localization and regional feature extraction, but their inherently local receptive fields limit the capture of long-range temporal coherence, particularly in low-frame rate cine MRI or sparsely reconstructed 4D CT [4, 23]. Recurrent architectures partially address this limitation by explicitly modeling temporal evolution; however, their performance degrades with long cardiac sequences and irregular temporal sampling, which are common in clinical imaging [12, 85]. Transformer-based models mitigate these constraints through global attention mechanisms and improved temporal context modeling, yet their reliance on large, diverse training cohorts poses challenges in single-center or limited-data cardiac studies [19, 79]. These architecture-specific limitations illustrate why purely data-driven models may produce temporally inconsistent or physiologically implausible motion estimates when applied to clinical cardiac imaging. Incorporating basic biomechanical or anatomical constraints into the learning process offers a practical way to stabilize motion estimation and improve robustness across varying image quality, temporal resolution, and imaging modalities[16, 86].

These architectural trade-offs are reflected in the representative studies summarized in Table 4. For instance, while CNN-based frameworks consistently achieve high spatial accuracy for segmentation (with Dice coefficients often exceeding 0.90, e.g., [29, 58]), their sensitivity for detecting regional abnormalities can be notably lower (e.g., 63.2% in [179]). This disparity suggests that high spatial performance in static frames does not inherently translate to the mechanical or clinical robustness required for motion analysis. Conversely, architectures that prioritize temporal modeling, such as those used in MRI strain estimation [13, 180], demonstrate higher correlation with clinical ‘ground truth’ but often require more specialized, multi-center datasets to achieve generalizability. This failure to bridge the gap between ‘segmentation accuracy’ and ‘diagnostic sensitivity’ is a primary reason why many purely data-driven models remain in the research phase rather than transitioning to routine clinical bedside use.

### Spatiotemporal Constraints in Clinical Imaging

Beyond the logistical hurdles of cost and accessibility, achieving sufficient temporal and spatial resolution in imaging continues to pose fundamental technical challenges [194, 195]. Specifically, the ability to accurately capture cardiac WMAs is fundamentally constrained by the physics of the underlying imaging modality [196, 197]. In cine MRI and cardiac CT, apparent image sharpness can be misleading; since interpolation or zero-padding improves visual appearance without increasing true acquired spatial resolution, deformation analysis remains limited by the native voxel size [196]. Furthermore, ultrasound-based modalities face a fundamental trade-off: high temporal resolution (frame rate) often necessitates a reduction in spatial line density or field of view. Insufficient spatial detail degrades endocardial and epicardial border delineation, particularly in 3D echocardiography or fetal imaging where small chamber sizes amplify the impact of voxel volume averaging [147, 198]. Temporal resolution plays a dominant role, as inadequate sampling smooths rapid mechanical events and biases peak strain and strain rate measurements [196, 199]. For instance, a temporal resolution less than 30 frames for strain or 50 frames for strain rate can lead to inaccurate peak systolic quantification [199]. Crucially, in cine MRI and cardiac CT, the number of reconstructed frames can be misleading; the true temporal resolution is governed by the data acquisition window. If this window is too wide, rapid mechanical transitions are effectively ‘motion-blurred’ regardless of the final reconstructed frame rate [196]. In the context of DL, these modality-specific constraints introduce artifacts, such as motion blur or aliasing, that must be accounted for during model training to ensure robust feature extraction across varying acquisition protocols.

### Challenges in DL Interpretability, Data Scarcity, and Clinical Translation

Some DL-based segmentation frameworks have begun to demonstrate partial integration into clinical workflows. However, these implementations are largely confined to volumetric or structural segmentation tasks, whereas comprehensive regional deformation and strain analysis remain predominantly research-oriented [181, 200].

DL models are sometimes viewed as “black boxes”, raising concerns about interpretability and clinical trust. They require large, high-quality, and well-annotated datasets for training, yet the availability of such datasets is limited [201, 202]. For example, studies have reported difficulties such as blurred boundaries between trabeculation and myocardium [184], atrial inclusion in basal slices with intensities similar to the LV [203], and challenges in epicardial extraction due to similar intensity with surrounding tissues [204].

In addition, most methods struggle to handle noisy or complex images, and their predictions remain largely limited to wall motion without extending to tissue characterization or functional properties. For instance, even clinically validated DL frameworks (such as [190]) that have strong generalizability across standard and portable ultrasound systems, remain restricted to binary detection of wall-motion abnormalities and retrospective validation. This shows the need for prospective and explainable modeling approaches.

Another important limitation is the scarcity of diverse datasets that capture anatomical variations, demographic differences, and pathological heterogeneity, which undermines generalizability [161].

Moreover, performance may vary across data acquired from different vendors, reflecting the lack of standardization in imaging protocols and model design. Bias introduced by vendor-specific image quality or clinical characteristics of the training cohort can compromise model robustness. As shown in Table 4, the lack of external validation across diverse cohorts remains a primary barrier to clinical adoption. Consequently, many models lack external validation, particularly prospective clinical trials, which hinders their translation into real-world practice.

## Conclusion

The prediction of cardiac wall motion has evolved from traditional imaging and computational approaches toward advanced machine learning frameworks capable of extracting complex spatiotemporal features with high accuracy. DL models such as CNNs, RNNs, and transformers have demonstrated significant potential in automating segmentation, motion estimation, and detection of regional wall motion abnormalities across CT, MRI, and echocardiographic datasets. While 4D CT and cine MRI have dominated research on DL-based wall-motion quantification, their routine clinical adoption remains limited. Most current applications are still developed and evaluated in academic or research settings, reflecting challenges related to data heterogeneity, model generalizability, and regulatory approval. In cardiac MRI, several DL-based segmentation frameworks have begun to show integration into clinical workflows, yet these advances primarily concern volumetric or structural segmentation rather than full regional deformation analysis. By contrast, echocardiography, with its real-time acquisition has achieved broader clinical implementation of DL-based functional assessment. In this context, rather than viewing CT, MRI, and echocardiography as competing modalities, emerging evidence supports multimodal DL pipelines in which CT and MRI provide high-fidelity geometric and strain supervision, while echocardiography serves as the primary deployment modality. Beyond functional assessment, recent advances indicate a shift toward prognostic modeling, where DL-derived motion representations inform clinical outcomes and risk prediction through the integration of motion dynamics with survival analysis. Despite these advances, important challenges remain, including the need for large and diverse annotated datasets, limited interpretability of DL models, and gaps in external and prospective clinical validation. Addressing these limitations will be crucial for translating current research into robust and generalizable tools that can be integrated into routine clinical workflows. Continued interdisciplinary collaboration between engineers, clinicians, and data scientists will be essential to build reliable cardiac wall motion simulators, ultimately supporting early diagnosis, personalized treatment planning, and improved cardiovascular care.

## List of Symbols

AUC: Area Under the Curve
CNN: Convolutional Neural Network
CT: Computed Tomography
DENSE: Displacement Encoding with Stimulated Echoes
DL: Deep Learning
ECG: Electrocardiography
FT: Feature Tracking
GCS: Global Circumferential Strain
GLS: Global Longitudinal Strain
GRS: Global Radial Strain
HFpEF: Heart Failure with Preserved Ejection Fraction
LSTM: Long Short-Term Memory
LV: Left Ventricle
LVEF: Left Ventricular Ejection Fraction
LVESV: Left Ventricular End-Systolic Volume
ML: Machine Learning
MRI: Magnetic Resonance Imaging
RMSE: Root Mean Square Error
RNN: Recurrent Neural Network
STE: Speckle Tracking Echocardiography
TDI: Tissue Doppler Imaging
WMA: Wall Motion Abnormality
